# A System to Track Stent Location in the Human Body by Fusing Magnetometer and Accelerometer Measurements

**DOI:** 10.3390/s23104887

**Published:** 2023-05-19

**Authors:** Yifan Zhang, William W. Clark, Bryan Tillman, Young Jae Chun, Stephanie Liu, Sung Kwon Cho

**Affiliations:** 1 Mechanical Engineering and Materials Science Department, University of Pittsburgh, Pittsburgh, PA 15261, USA; 2 Vascular Surgery, The Ohio State University, Columbus, OH 43210, USA; 3 Industrial Engineering Department, University of Pittsburgh, Pittsburgh, PA 15261, USA

**Keywords:** magnetic field, location tracking, magnetometer, stent guidance, sensor fusion

## Abstract

This paper will introduce a simple locating system to track a stent when it is deployed into a human artery. The stent is proposed to achieve hemostasis for bleeding soldiers on the battlefield, where common surgical imaging equipment such as fluoroscopy systems are not available. In the application of interest, the stent must be guided to the right location to avoid serious complications. The most important features are its relative accuracy and the ease by which it may be quickly set up and used in a trauma situation. The locating approach in this paper utilizes a magnet outside the human body as the reference and a magnetometer that will be deployed inside the artery with the stent. The sensor can detect its location in a coordinate system centered with the reference magnet. In practice, the main challenge is that the locating accuracy will be deteriorated by external magnetic interference, rotation of the sensor, and random noise. These causes of error are addressed in the paper to improve the locating accuracy and repeatability under various conditions. Finally, the system’s locating performance will be validated in benchtop experiments, where the effects of the disturbance-eliminating procedures will be addressed.

## 1. Introduction

For soldiers injured on the battlefield, effective and prompt control over internal hemorrhage, particularly for major vessel trauma, is essential for their survival. According to a research study, 20% of mortalities during a war happen before the soldiers can reach treatment facilities, and the major cause in this group is hemorrhage, which accounts for over 50% of the deaths [1]. Many hemostasis strategies such as drugs or compression bandages are introduced in [2]. Among all the approaches, a retrievable rescue perfusion stent can achieve hemostasis most swiftly, which makes it very promising for the extremely dangerous condition in which the wound occurs in the aorta deep inside the torso [3]. The cylindrical stent is covered with an impermeable Polytetrafluoroethylene (PTFE) layer. When deployed into the aorta, the stent expands to support the PTFE layer to cover the wound in the vessel, thereby creating an effective second interior artery wall, which has been shown to reduce 93% of hemorrhage within 10 s, while the blood can still flow within. The structure of this stent and its performance in hemorrhage control in an animal test is introduced in [3,4]. As an emergency aid device for when the location of the wound cannot be accurately visualized, the stent may be designed to be long enough to cover a large portion of the artery; however, such a design can cause side effects since all branch vessels along the artery are occluded by the stent. For example, when the wounded major artery is the celiac trunk, which contains branches of the hepatic, splenic, and left gastric arteries, these branches may become blocked by the stent, resulting in lactate acidosis, sepsis, renal insufficiency, and even fatal complications [5].

**Figure 1:**
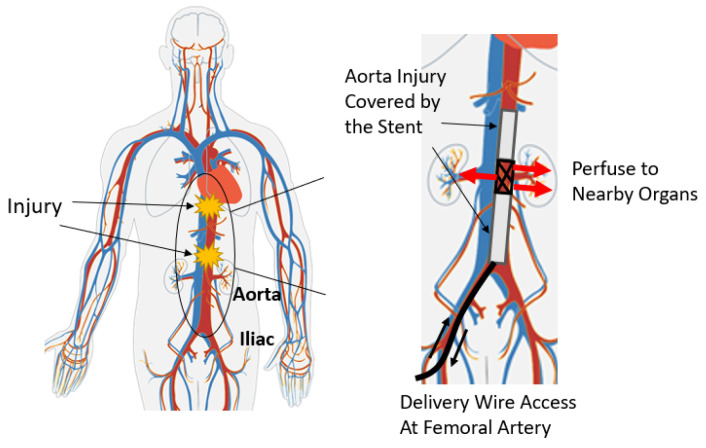
The structure of the impermeable stent and how it achieves hemostasis at the desired location in the aorta [3]. Reprinted/adapted with permission from Ref. [6], 2010, Wikimedia Commons.

To avoid occlusion where vital branch arteries are located, the stent design presented by Chun [3] has a portion uncovered by the PTFE layer. In practice, the procedure for placement of the perfusion stent and accompanying sensor first involves inserting it into the body through an incision on the iliac artery and then pushing it cranially in the torso by a guide wire. While advancing intermittently, the uncovered part of the stent must be matched with the branch arteries to keep the branching routes open. The celiac trunk generally lies within 1–3 cm of the xiphoid bone, which can be felt externally on the body. Therefore, the xiphoid bone is regarded as the landmark of the target location. Location measurement of the device inside the body with respect to this landmark enables proper placement of the stent, which leaves the branch vessels free for blood flow. This desired function of the stent is illustrated in Figure 1.

In the application described, rapidly deploying the stent is critically important for stabilizing the patient. Key requirements are minimal hardware so that the stent locating system can be quickly set up, and the locating accuracy should be less than 0.5 cm at the target location. These two requirements are the motivating factors for the system described in this paper. While there are existing locating systems for various surgical use cases, even ones that utilize a magnetic field as in this work, none meet the simplicity, rapid deployment, and calibration-free demands that are called for in this application.

Current object locating techniques for surgical application can be achieved in several different ways. The first method is to use an imaging system that shows the features of interest inside the body. Imaging modalities include fluoroscopy, computed tomography, magnetic resonance imaging, and ultrasound. Because of their clarity and reliability, image-based systems are widely implemented in hospitals [7]. However, obvious drawbacks of these systems are that they are not portable and are inconvenient to use, especially for emergent situations like on the battlefield. Therefore, some portable systems that can be rapidly deployed have been developed as alternatives.

Bianchi has made a comprehensive review of currently available localization approaches in the field of bioengineering. Among all approaches, magnetic trackers have been used in applications such as tracking eye and head motion [8,9]. The magnetic trackers always include a magnetic source and several sensors, which are arranged around the targeted objects. The target objects (eyes and head) are in the open air, which allows a comparably large size and number of applied sensors and makes camera trackers feasible as the object is in the line of sight. Furthermore, the environment for these devices is typically controlled and the time of setup is not limited. In this situation, a magnetic tracker could reach an accuracy of 0.15 mm and 1${}^{\circ }$ in translation and rotation. For in-torso surgeries where the mentioned trackers are not viable, smaller and reliable magnetic trackers have been used in many studies [10,11]. For these systems, the sensor can work in vivo and achieve locating accuracy of 3 mm. Compared with other waveforms, the magnetic field can penetrate non-metal substances with little distortion and attenuation and with minimal risk to the body [12]. One drawback of the current magnetic trackers is their susceptibility to decreases in measurement accuracy under varying environments. For example, the location measured at the corner of walls can be different from the measurement collected at the center of the room by up to 2 cm. This error is due the distortion of the reference magnetic field by disturbing magnetic sources, and the error increases when the sensor disturbance distance decreases [13]. For the primary application of interest in this paper, where the sensor is to be used on the battlefield in an urgent trauma situation, there is no certainty that the procedure will be performed in a disturbance-free environment, and the conditions may be cramped, moving, vibrating, and have many surrounding obstacles, so the system described in this paper emphasizes disturbance rejection.

Based on the type of EMF source, current trackers can be classified into two categories. The first category applies high-frequency alternating current (AC) to generate a high-frequency electromagnetic field (EMF) on multiple coils that are arranged in a certain pattern. For a sensing coil near these active coils, a current will be induced, whose amplitude is related to its relative location with respect to the EMF sources [13]. This theory has been widely applied in commercial products such as Aurora and Polhemus [14,15].

The second category applies permanent magnets or electromagnets powered by direct current (DC) to generate a static magnetic field (SMF). The sensor applied in this type of system is usually a magnetometer that measures magnetic flux density. Compared with the AC locating approach, this method is relatively simple and cheap to implement. The SMF can be applied to achieve locating tracking in two approaches. First, when the sensor-magnet distance is much larger than the magnet’s size, a cylinder permanent magnet can be simulated by a dipole model. Multiple sensors are arranged around the magnet. The location and heading angle of the permanent magnet with respect to the entire sensor array can be solved by an optimization algorithm [16]. This approach can be further improved by implementing dynamic sensors to measure the target acceleration, then fusing the magnetic and dynamic measurements with Kalman filter to give a more accurate result [17]. For our application, the magnet’s size is restricted by the artery’s diameter (3 mm). Such a small magnet makes most commercial products unfeasible, and could potentially make the locating accuracy drop due to the weak strength. In an alternate configuration, the magnetic source is placed outside while the sensor goes into the body. The advantage is that the strength of the magnet can be adjusted over a wide range, allowing flexibility in the measurement process. The second approach requires the knowledge of the magnetic field created by a reference magnet, in which the magnetic flux density vectors are paired with the location coordinate and recorded in advance as a lookup table. New measurements from unknown locations are then searched in the lookup table to find their closest matches; thereby, the related locations can be determined. This theory is introduced in [18]. Similar to the EMF approach, the SMF locating approach is also susceptible to the background magnetic distortion. Disturbing SMF sources such as the geomagnetic field and ferrous materials nearby have a greater impact on this locating approach. In the work from [19,20], the background disturbance is regarded as a constant value over the test range. However, the magnetic disturbance can vary with time and space in practice, which makes an initial compensation less reliable with increasing time and movement distance.

This paper applies the second locating approach with an SMF source and aims at reaching a higher accuracy and better robustness by rejecting the varying disturbance. To achieve this, several new contributions are made. A new model that describes all major magnetic disturbances is developed, and a corresponding solution method will be given. In addition, an inertial sensor which can measure the heading direction will be fused with the magnetic measurement to improve the system reliability. At last, the accuracy and robustness of the locating system will be evaluated in experiments where the sensor’s working condition simulates a real surgery.

## 2. Location Measurement Procedures

### 2.1. Locating Principle with Magnetic Field

The magnetic locating approach uses one cylindrical magnet as the source of the reference magnetic field. The center of the magnet is treated as the origin of an orthogonal coordinate system, which can be called the magnet frame. In practice, the magnet is placed on the patient’s chest right above the target location. The locating system starts functioning as soon as the surgeon turns on the power supply. When the stent with the attached detector is inserted as usual, its location can be expressed by three-dimensional coordinates in this magnet frame by relating the measurement to its location in the theoretical reference magnetic field. A grid of magnetic field vectors surrounding the reference magnet is computed a priori and stored. Upon obtaining a measurement vector during stent deployment, the reference grid is searched to find the closest match, which will be taken to be the measured location of the sensor. With the use of the theoretical magnetic field reference described here and the noise-canceling approach described in the next section, the system can avoid going through a time-consuming calibration procedure before use. In short, the system can be set up within seconds then continuously provide the real-time position information about the stent’s location with necessary accuracy as the stent is being deployed.

**Figure 2:**
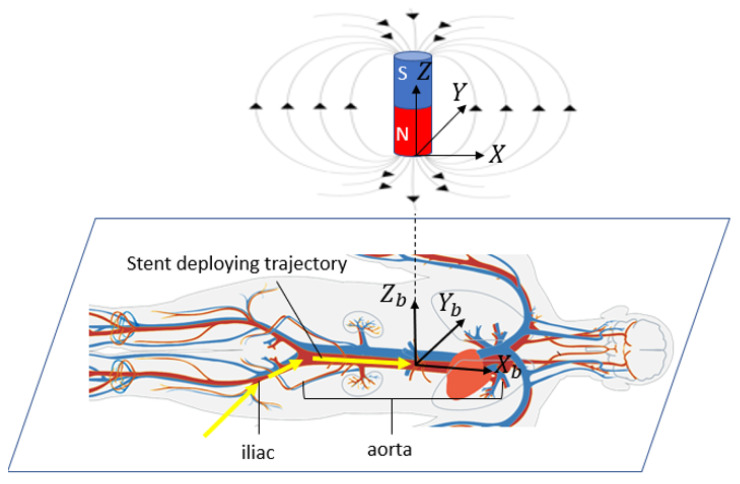
The reference magnetic field and the detector trajectory in the torso. Reprinted/adapted with permission from Ref. [6], 2010, Wikimedia Commons.

The schematic of this method in surgical application is shown in Figure 2, where a body is shown as it would be in practice lying on a flat surface. A body coordinate system is shown with $X_{b}$ aligned with the aorta (along the length of the torso). $Y_{b}$ is transverse to the aorta and in the horizontal plane, and $Z_{b}$ is vertical (aligned with gravity). The origin of the body coordinate system is assumed to be in the center of the aorta, directly under the xiphoid, though in general it could be located at any point relative to any bony landmark. The goal in this application is to locate a specific portion of the perfusion stent (e.g., the leading edge) in the body coordinate system. Figure 2 also shows an electromagnet located outside the body with a magnet coordinate system, with its axes of X,Y,Z. The algorithm, as described later, will determine the sensor location within the range of the electromagnet, expressed in the magnet coordinate system. If the magnet coordinate system is aligned with and at a known location relative to the body coordinate system, then one can transfer a known location in the magnet frame to the body frame. This relative alignment and positioning is achieved by first placing the electromagnet on the body in a known position and orientation relative to an appropriate landmark. In this study, the placement of the magnet is at the origin of the body frame, which is right above the xiphoid bone. For a cylindrical magnet, the radial component of the magnetic field is symmetric. Therefore, the *X* axis in the magnet frame is taken to be aligned to be parallel with $X_{b}$ in the body frame. In addition, the *Z* axis is aligned vertically in space, thereby fixing the direction of the *Y* axis. In this study, the destination of the sensor is directly under the xiphoid bone, in the aorta, though it could be set at any other location relative to the electromagnet within the measurable field. After the stent enters the aorta, the trajectory can be approximated as a straight line.

**Figure 3:**
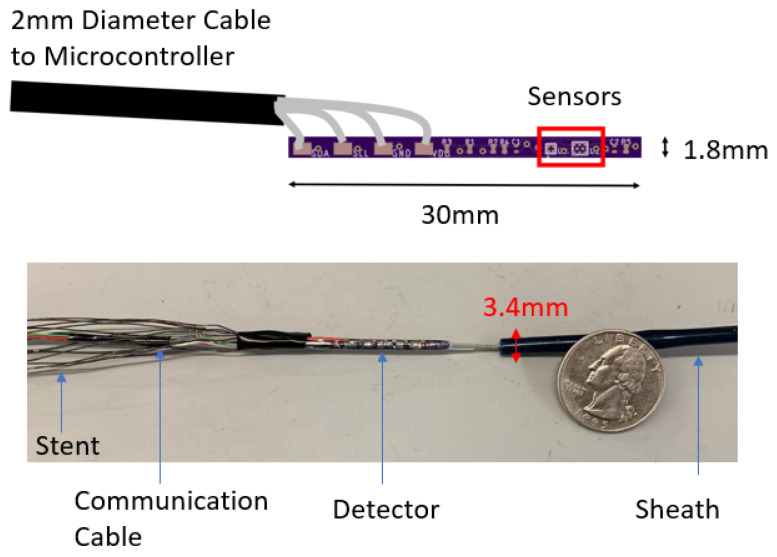
The schematic and the fabricated detector with the sheath and stent.

The goal of the locating system is to track the position of the sensor (and therefore the stent) along the aorta and report its position in the body frame. The detector consists of one triple-axis accelerometer (MXC400xXC) and one triple-axis magnetometer (MMC5603NJ) soldered onto a printed circuit board (PCB). The schematic and the fabricated sensor are shown in Figure 3. The overall size of this detector has length of 30 mm and diameter of 1.8 mm. The PCB is attached to the leading edge of the stent, which is also attached to a guide wire used for insertion into the body. The relative position of the PCB to the stent is recorded. Because a guide wire is needed for stent deployment, the additional wires for communication with the sensor do not pose a problem, as they can be attached to the guide wire. This eliminates the need for extra circuitry with the sensor that might otherwise be needed for wireless communication. The stent is made from Nitinol, whose magnetic permeability and susceptibility are both low enough to be ignored [21]. All implantable parts are contained inside a sheath, which has a diameter of 10 Fr (3.4 mm). When the sensor reaches the target location in vivo, the sheath will be removed so that the stent will expand to cover the trauma on the vessel.

**Figure 4:**
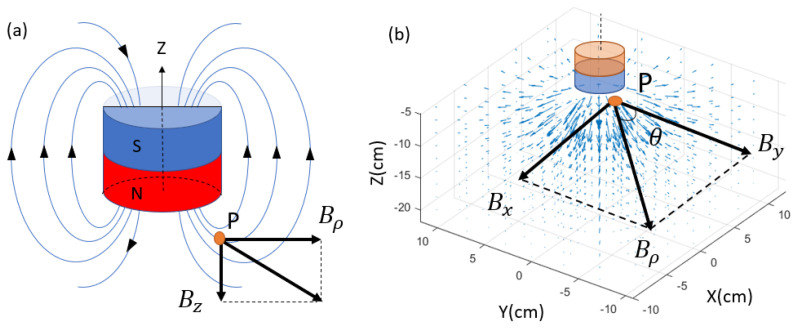
Magnetic field components for a cylindrical magnet. (**a**) The radial $B_{\rho }$ and axial $B_{z}$ components in a vertical plane that pass through the cylinder center. (**b**) Decomposition of $B_{\rho }$ in a horizontal plane.

The reference magnetic field source in this research is provided by a cylindrical magnet, the most common shape for both permanent magnets and electromagnets. There are several analytical approaches to generate the magnetic field spatial distribution. One common approach is to simulate the cylinder magnet by a dipole model [8,19]. This approach requires the magnet size to be small compared to the target–sensor distance. If the magnet has a significant size, as in this work, another model that is based on the Biot–Savart law can be applied. This model simplifies the cylinder magnet as two parallel planar coils, with current flowing in the same directions. In this research, the height of the cylindrical magnet is L=5 cm, and the radius a=3.75 cm. In any plane that passes through its central axis, the magnetic flux density vector can be decomposed into an axial part $B_{z}$ and a radial part $B_{\rho }$, which are orthogonal and can be obtained by Equations (1) and (2) [22]. These two equations are generally used with SI units and provide the flux density in Tesla units (T). In this paper, some quantities are more convenient to be measured using CGS units, such as the flux density, which is measured in Gauss (G), and the system dimensions are measured in centimeters (cm). The $\mu_{0}M$ term will be later represented by a dimensionless coefficient. By rotating $B_{\rho }$ with angle θ around the Z axis, $B_{\rho }$ is decomposed into $B_{X}$ and $B_{y}$ through Equations (3) and (4), while $B_{z}$ is unchanged with θ. The spatial distribution of the flux density vector $\vec{B}$ is extended into the 3D coordinate system, as shown in Figure 4. This model has been validated by comparing the simulation to the actual value measured by the sensor. However, it is worth mentioning that this model is still not a perfect simulation of a real electromagnet because it ignores factors such as the ferromagnetic nucleus and the boundary condition between the coil and the shell. A more comprehensive model is an objective for our future research.

(1) $$B_{z}=\frac{\mu_{0}}{4\pi }M\int_{0}^{2\pi }\int_{0}^{a}\frac{R\left(L/2-z\right)}{{(R}^{2}+{\left(L/2-z\right)}^{2}+\rho^{2}-2R\rho \cos{\Phi )}^{3/2}}+\frac{R\left(L/2+z\right)}{{(R}^{2}+{\left(L/2+z\right)}^{2}+\rho^{2}-2R\rho \cos{\Phi )}^{3/2}}dRd\Phi$$

(2) $$B_{\rho }=\frac{\mu_{0}}{4\pi }M\int_{0}^{2\pi }\int_{0}^{a}\frac{R\left(\rho -R\cos\Phi \right)}{{\left(R^{2}+{\left(L/2-z\right)}^{2}+\rho^{2}-2R\rho \cos\Phi \right)}^{3/2}}+\frac{R\left(\rho -R\cos\Phi \right)}{{\left(R^{2}+{\left(L/2-z\right)}^{2}+\rho^{2}-2R\rho \cos\Phi \right)}^{3/2}}dRd\Phi$$

(3) $$B_{x}=B_{\rho }cos\theta$$

(4) $$B_{y}=B_{\rho }sin\theta$$

In Equations (1) and (2), $\mu_{0}$ is the constant of air permeability. The magnetic permeability of all human body components can be approximated by the permeability of water, whose difference to air permeability is negligible [7]. Therefore, it is valid to treat the permeability as a static parameter. *M* is a constant characteristic parameter of a permanent magnet called magnetization that describes the magnetic moment in the unit volume [23].

Once the predicted field pattern is known, the next step is to locate new measurements in this pattern. In this work, a nearest neighbor algorithm is used. Assume there are reference points with total number of *a* distributed in the SMF pattern. The reference points are indexed by 1,2,3,…,a. For every arbitrary point indexed with *i*, the location coordinate $\left(X_{i},Y_{i},Z_{i}\right)$ is related to the local magnetic field prediction $\left(B_{xi},B_{yi},B_{zi}\right)$ using Equations (1)–(4). The field will be measured with a three-axis magnetometer. When a measurement $\left(B_{MX},B_{MY},B_{MZ}\right)$ is taken from the sensor at an unknown location, the magnitude of the difference $D_{i}$ between the measurement and reference values is calculated by Equation (5). Then an exhaustive calculation of $D_{i}$ for i=1,2,3,…,a is performed for all reference points. The desired location of the sensor can be determined by the reference location where the minimum of $D_{i}$ occurs. The search is computed with the Python scikit-learn module, which can accomplish the task with excellent speed and accuracy [24].

(5) $$D_{i}=\sqrt{{\left(B_{MX}-B_{Xi}\right)}^{2}+{\left(B_{MY}-B_{Yi}\right)}^{2}+{\left(B_{MZ}-B_{Zi}\right)}^{2}}$$

In the surgical application in which the magnet is placed right over the body landmark with axes aligned with the patient’s body in the previously introduced way, the sensor’s horizontal distance to the destination along the aorta is represented by the *X* axis coordinate. The measurement in this dimension is the most valuable one for the application in this paper. The *Y* and *Z* axes readings, which represent the side and vertical location offsets from the magnet center, will not change drastically as the stent moves along the aorta.

### 2.2. External Magnetic Disturbance Cancellation

The working principles of the location methods introduced above are valid under ideal conditions without mentioning noise and interference. In practice, various sources of magnetic interference, including the earth’s magnetic field, a metal surgical bench, or a bullet fragment inside the patient’s body, are all possible sources of error for magnetic-based locating systems [25]. Mathematically, the effect of all the disturbances is expressed by Equation (6) [26]. In this equation, ${\vec{B}}_{m}$ is a 3×1 vector of the three-axis sensor’s actual measurement under interference, and ${\vec{B}}_{r}$ is a 3×1 vector of the true magnetic field without interference at the same location. W and $\vec{V}$ are accuracy-degrading factors. In many studies, W is called the soft iron effect, whose main cause is the soft iron materials around the sensor that could change the nearby magnetic permeability. The factor $\vec{V}$ is called the hard iron effect, which comes from all permanently magnetized components fixed around the magnetometer. Besides the components soldered on the PCB, interference could also come from the stent and the guide wire, which may be made from magnetic material. In addition, the magnetometer may have manufacturing bias or temperature-induced bias. A more comprehensive analysis for all causes of error is introduced in [27]. Inherent sensor biases are typically removed by a calibration procedure before the actual experiment [16]. The calibration algorithm requires the sensor to be held at a fixed location in a homogeneous field and rotated randomly to *k* directions while ensuring adequate coverage of all octants. The solution of $\vec{V}$ can be obtained when the overall difference from all *k* measurements to the static magnitude reaches the minimum [26]. In addition, this method enables the true sensitivity values for each axis (in matrix W) to be determined. A more reliable calibration approach by creating an uniform magnetic field is introduced in [17]. The proposed method in this paper does not address these biases but focuses on external disturbances to the measured magnetic field.

(6) $${\vec{B}}_{m}=W{\vec{B}}_{r}+\vec{V}$$

(7) $${\vec{B}}_{m}=WI{\vec{B}}_{0}+\vec{V}+{\vec{V}}_{per}+I{\vec{V}}_{soft}$$

**Figure 5:**
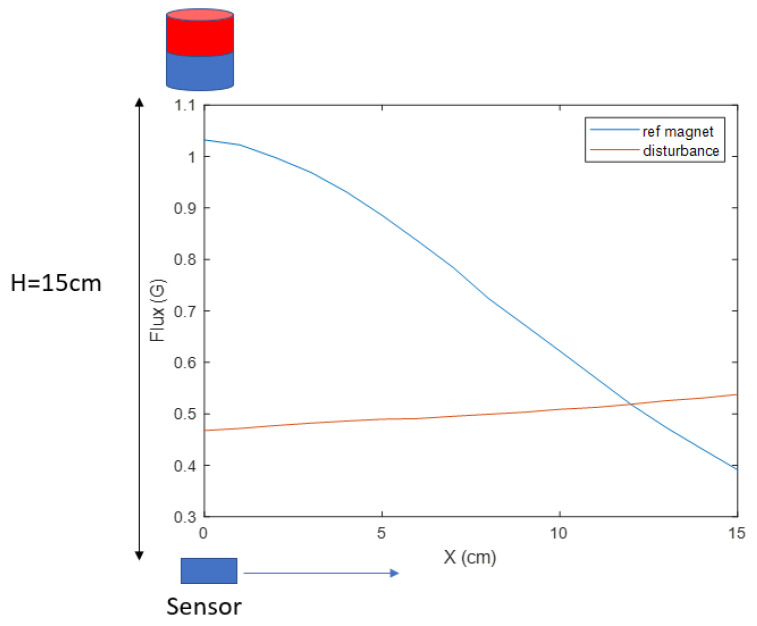
The magnetic field measurement of the permanent reference magnet and the disturbance.

An example experiment was carried out to measure the range of $|{\vec{B}}_{r}|$ and $|\vec{V}|$ using a Neodymium cylinder magnet (one of the most powerful magnet materials commercially available [28]) with a diameter of 0.8 cm and height of 1 cm as the magnetic source. The magnet is placed 15 cm above the sensor. In the horizontal range of 15 cm, the magnetic measurement in shown Figure 5, where the reference magnetic strength $|{\vec{B}}_{r}|$ varies from 1.03 to 0.39 G, and the disturbance $|\vec{V}|$ varies from 0.46 to 0.53 G. In this test, no ferrous objects exist around the system, which makes the geomagnetic field the only source of $\vec{V}$. The ratio of the $|\vec{V}|$ range to the $|{\vec{B}}_{r}|$ range is 0.11 in this test. This ratio can be reduced by applying a more powerful magnet source. However, the constant disturbance is not a reasonable assumption for this application because the reliability of our magnetic locating approach requires the measurement of the reference magnetic field to be as accurate as possible. The locating accuracy drops as interference strength increases, which cannot be controlled on the battlefield, where multiple disturbances may exist. Therefore, an electromagnet was chosen as the desired source, whose strength of the generated magnetic field is proportional to the applied current *I*. The exclusive parameter *M* of a permanent magnet in Equation (1) and (2) is replaced by $I{\vec{B}}_{0}$, where ${\vec{B}}_{0}$ is a unit magnetic field strength vector when I=1A. The actual value of ${\vec{B}}_{0}$ is still hard to measure directly. To find its true value for a certain electromagnet, first, the spatial distribution is generated by assuming I=1A and $\mu {\vec{B}}_{0}=1$. Next, measurements are collected along a straight trajectory that passes through the center of the magnet. The ratio between the measurements and the analytical model at corresponding locations in each axis is calculated and then averaged, which is denoted by $\vec{C}$. When this is tested in advance for a chosen electromagnet, the actual value of the SMF can be expressed by replacing the μM term in Equation (1) and (2) by $I\vec{C}$.

When an electromagnet is used as the SMF source, a better simulation of the interference is developed through Equation (7). The interference sources are classified into two categories; the first source is from the near-sensor range, which includes sensor manufacturing variance, the materials on the printed circuit board (PCB) onto which it is soldered, and the stent wires. After the detector is fabricated, the effect from these components will be constant; these components are expressed by W and ${\vec{V}}_{per}$. The second source includes the interference in the background; the additional term $\vec{V}$ corresponds to the disturbance that is invariant with respect to time but variant with respect to location. $\vec{V}$ can be removed by taking advantage of the electromagnet. When a switching relay is turned on and off in the electromagnet circuit, the current will switch between a constant amplitude *I* and 0. At a given measurement location, when the switch is turned off, the background magnetic field is measured and denoted as ${\vec{B}}_{1}=\vec{V}+{\vec{V}}_{per}$, then the current is turned on and the new measurement is denoted as ${\vec{B}}_{2}$ (Equation (7)), which includes background vector ${\vec{B}}_{1}$ superposed on the desired magnetic reference vector. Therefore, the disturbance can be removed from the desired measurement by ${\vec{B}}_{2}-{\vec{B}}_{1}$. This switching process is repeated frequently while the detector is moving so that it can deal with an interference that varies with location. Its effectiveness requires that ${\vec{B}}_{1}$ and ${\vec{B}}_{2}$ are measured at a stationary location, which means the current switching should be frequent enough so that the displacement between two measurements in a dynamic process is negligible. In addition, the electromagnet should be able to generate a constant ${\vec{B}}_{0}$ throughout the measurement. These factors will be discussed in the later section about the experiment.

The soft iron effect term W can be solved by calibration in advance or approximated as an identity matrix in many studies [19]. The term $I{\vec{V}}_{soft}$ is added in Equation (7) to represent soft iron objects that are not fabricated around the sensor. It is an assumption that these interferences are caused by objects such as the bullet or shrapnel shell that are magnetized by the source magnet. Therefore, $I{\vec{V}}_{soft}$ is a highly random term that depends on the number and magnetic susceptibility of all objects in the surrounding regions. Their effect will appear and disappear as the electromagnet source is switched on or off, and the amplitudes are proportional to the source SMF strength. Assuming W is an identity matrix and the hard iron interference has been successfully removed by the approach introduced before, the measurement error can be expressed by Equation (8). At a stationary location, by adjusting and measuring the current amplitude and then correspondingly varying ${\vec{B}}_{m}$, the slope of the *I*-$|B_{m}|$ curve is the magnitude of $|{\vec{B}}_{0}+{\vec{V}}_{soft}|$. This approach cannot directly cancel the soft iron object disturbance, but it can show how serious the soft iron effect from potential interfering sources is.

(8) $${\vec{B}}_{m}=I\left({\vec{B}}_{0}+{\vec{V}}_{soft}\right)$$

### 2.3. Tilt Angle Compensation

**Figure 6:**
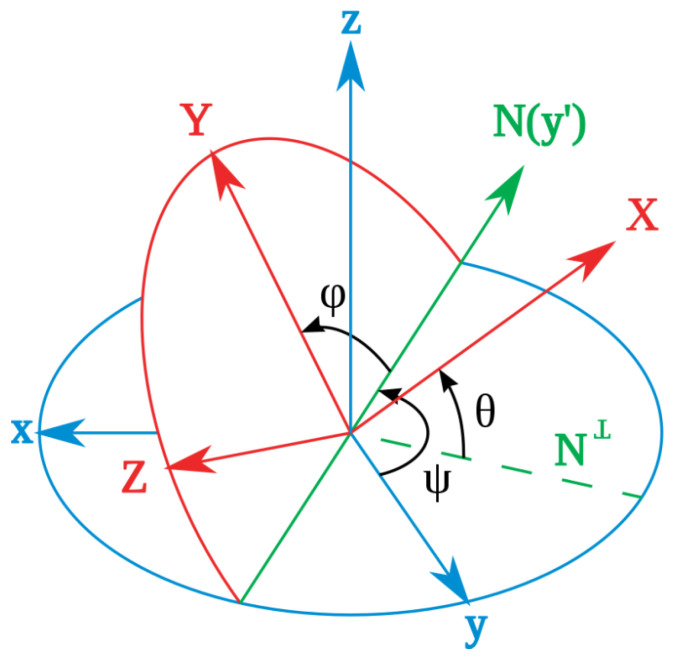
Definition of roll(φ), pitch(θ), yaw(ψ) angles. Reprinted/adapted with permission from Ref. [29], 2009, Wikimedia Commons.

In the process of deploying the sensor inside the artery, the sensor’s linear translation may be accompanied by rotation. To obtain the closest matching point with a given measurement, the sensor’s local coordinate frame always needs to be kept aligned with the magnet frame (this assumes a straight-line path of the aorta in the present application). Otherwise, if the sensor rotates with respect to the magnet frame, then the locating method that finds the matching magnetic field point is invalid. As is shown in Figure 6, for an arbitrary orientation of the sensor in the body, the fixed magnet frame (x,y,z) can be rotated to the direction of the sensor frame (X,Y,Z) by first rotating around the *Z* axis with angle ψ, then rotating around the *Y* axis with angle θ, and finally rotating around the *X* axis with angle φ. These three angles are Euler angles and are usually called yaw, pitch, and roll. In this application, the stent is inserted through a non-solid guiding wire to move inside the thin and straight artery. This feature makes the detector rotation most likely to happen around the *X* axis, the roll angle. Therefore, the roll angle measurement is the most important secondary measurement in this system.

To measure the Euler angles, additional sensors should be added to the detector. There are two types of sensors that can achieve this. The first one is the gyroscope that measures the angular velocity, which can be continuously integrated to obtain the Euler angles’ change [30]. This method is not applied in this paper to avoid possible direction measurement error that grows with time due to the integration of gyro drift. Instead, an algorithm introduced by Ozyagcilar that uses an accelerometer is applied [31]. Before the sensor is used in practice, calibration is necessary for an accelerometer. The accelerometer measurement error can be simplified as a constant bias in the way expressed by Equation (9), where ${\vec{a}}_{m}$ is the measurement, ${\vec{a}}_{r}$ is the real acceleration, and $\vec{b}$ is the bias error.

(9) $${\vec{a}}_{m}={\vec{a}}_{r}+\vec{b}$$

To solve the *X* axis component of $\vec{b}$, which is denoted as $b_{x}$, the X axis of the sensor should be first aligned vertically, with the X axis reading denoted as $a_{x1}$. Then the sensor should be flipped upside down, with the X axis rotated 180°, with the second reading denoted as $a_{x2}$. $b_{x}$ can be obtained as $b_{x}=\left(a_{x2}+a_{x1}\right)/2$. The other two components can be obtained in the same way. The accelerometer signal contains both a static component (acceleration of gravity, 1 g) and a dynamic component (acceleration of the sensor as it is moved). While the sensor is approximately in its static state, or during very slow movements, the dynamic component is minimal and the measurement only consists of the gravity acceleration. However, the stent deployment process is a dynamic process, where the sensor is repeatedly moved, then stopped, then moved again. Therefore, in practice the movement state is determined as either static or dynamic by setting $0.95\;g<|\vec{a}|<1.05\;g$ as the static acceleration threshold. When the static condition is satisfied, the gravity acceleration *g* in the magnet frame and the measurement $g_{p}$ in the sensor frame can be expressed by Equation (10). This equation shows how to mathematically convert from one frame to another frame through a certain rotation sequence in roll, pitch, and yaw. Even though pitch and yaw should have little variation in this application, the measurement approach will be introduced to make the theory comprehensive.

(10) $${\vec{g}}_{p}=R_{X}\left(\varphi \right)R_{Y}\left(\theta \right)R_{Z}\left(\psi \right)\vec{g}$$

(11) $$\vec{g}=\left(\begin{array}{c} 0\\ 0\\ g \end{array}\right){\vec{g}}_{p}=\left(\begin{array}{c} a_{x}\\ a_{y}\\ a_{z} \end{array}\right)$$

(12) $$R_{X}\left(\varphi \right)=\left[\begin{array}{ccc} 1 & 0 & 0\\ 0 & \cos\varphi & \sin\varphi \\ 0 & \sin\varphi & \cos\varphi \end{array}\right]$$

(13) $$R_{Y}\left(\theta \right)=\left[\begin{array}{ccc} \cos\theta & 0 & \sin\theta \\ 0 & 1 & 0\\ -\sin\theta & 0 & \cos\theta \end{array}\right]$$

(14) $$R_{Z}\left(\psi \right)=\left[\begin{array}{ccc} \cos\psi & \sin\psi & 0\\ -\sin\psi & \cos\psi & 0\\ 0 & 0 & 1 \end{array}\right]$$

It can be noticed that $R_{Z}\left(\psi \right)\vec{g}=\vec{g}$, which suggests the gravity acceleration measurement will not be affected by the change in ψ. In other words, ψ cannot be measured with gravity acceleration. Therefore, we can first solve φ and θ by substituting Equations (12) and (13) into Equation (10) and ignoring $R_{Z}$ to get the solution of roll and pitch by Equations (15) and (16). Note that for periods during which the measurement amplitude is not within the static range between 0.95g and 1.05g, the most recently found roll and pitch angles are used as approximations. As mentioned above, during stent deployment, these periods are intermittent and not problematic.

(15) $$\varphi =\tan^{-1}\left(-\frac{a_{y}}{a_{z}}\right)$$

(16) $$\theta =\tan^{-1}\frac{a_{x}}{\sqrt{a_{y}^{2}+a_{z}^{2}}}$$

To obtain the yaw angle ψ, the help of the geomagnetic field is needed, where 0° is defined as the direction of the geomagnetic north pole. Since the magnetometer and accelerometer are fixed on the PCB to keep the three axes orthogonal to each other, the rotation matrix of Equations (12) and (13) can be applied to rotate a random direction measurement of the geomagnetic field vector $B_{m}=\left[B_{mx},B_{my},B_{mz}\right]$ into a horizontal plane, where the new vector ${\vec{B}}_{p}$ has its *Z* axis component as 0. From its X-Y components, the yaw angle can be obtained by Equation (17) [31]. However, this approach is not reliable enough because the magnetic disturbance will be included in the measurement. Therefore, the yaw angle will be taken as a constant zero in this system.

(17) $$\psi =tan^{-1}\left(-\frac{B_{py}}{B_{px}}\right)=tan^{-1}\left(\frac{B_{mz}sin\varphi -B_{my}cos\varphi }{B_{mx}cos\theta +B_{my}sin\theta sin\varphi +B_{mz}sin\theta cos\varphi }\right)$$

Finally, when the Euler angles are solved, for a measurement ${\vec{B}}_{m}$ that is heading to a random direction, the tilt compensation can be applied with the following equation:

(18) $$\vec{B}=R_{x}^{-1}\left(\varphi \right)R_{y}^{-1}\left(\theta \right)R_{z}^{-1}\left(\psi \right){\vec{B}}_{m}$$

## 3. Experiment

### 3.1. Ideal Condition Test

This section will introduce the realization of in vitro experiments used to validate the system’s performance. All parts used in the system are listed in Table 1. The entire system is controlled by a Raspberry Pi, whose tasks include data collection, reference electromagnetic field control, location searching, and display of results. The suitably small magnetometer and accelerometer are wired to the Raspberry Pi through its I2C bus.

The electromagnet is powered by an external power supply and connected to the Raspberry Pi through a relay, which can be switched on and off to control the current flow. In the experiment, the current switching frequency is 5 Hz. A real electromagnet is expected to have dynamics that create a delay between the input power application and the resulting field response. The electromagnet used in the tests has a measured response time in the range of 0.04 to 0.08 s due to the RL time constant, which limits the frequency at which it can be switched. The cylinder magnet has a diameter of 7.5 cm and height of 5 cm and a maximum working DC voltage of 24 V, which can be provided by the DC power supply. The temperature of the electromagnet will increase with the working time. This will result in an increase in coil resistance and a decrease in current amplitude, which will proportionally change the field magnitude. Therefore, a current sensor is applied to measure the real-time current amplitude and to adjust the expected magnetic field accordingly. It should be noted that an alternate and perhaps simpler approach would be to use a constant-current power supply that would maintain a desired coil current and thus constant field. In addition, in practice, one could consider using an alternate electromagnet with a ferromagnetic (soft iron or ferrite) core, which could have multiple benefits, including possible reduction in the coil size (which may enable a dipolar approximation to the magnetic field model) and possibly a reduction in the power consumption that causes heating of the coils.

The assumed working space of the reference magnetic field pattern is a 40 cm × 40 cm × 20 cm rectangular space. This range is large enough to cover the area of interest on the patient’s body. It is important to note that the field is inhomogeneous and that inhomogeneity changes with respect to distance from the source, so one might consider creating a grid whose spacing varies accordingly (tighter spacing close to the source and wider spacing away from the source). In this paper, the spacing was uniform but close enough to obtain good results. Examples of the measured magnetic field are shown in later sections, but for an understanding of the system, in this range, the minimum amplitude of the SMF is 0.07 G and may increase to values as high as 3 G under ideal conditions. In comparison, field disturbances of 3–4 G may be observed. The model of the field is discretely sampled with a 0.2 cm resolution in each dimension to create the reference field, where the minimum absolute divergence is 0.025 G. The resolution of the chosen magnetometer is 0.06 mG, and the standard deviation of the static noise under the lab conditions tested is 1.3 mG. Therefore, the largest noise-to-signal ratio is 0.0186, which keeps the locating measurement reasonable for this test and makes it possible to create a new reference field with a smaller location resolution for further improvements.

**Figure 7:**
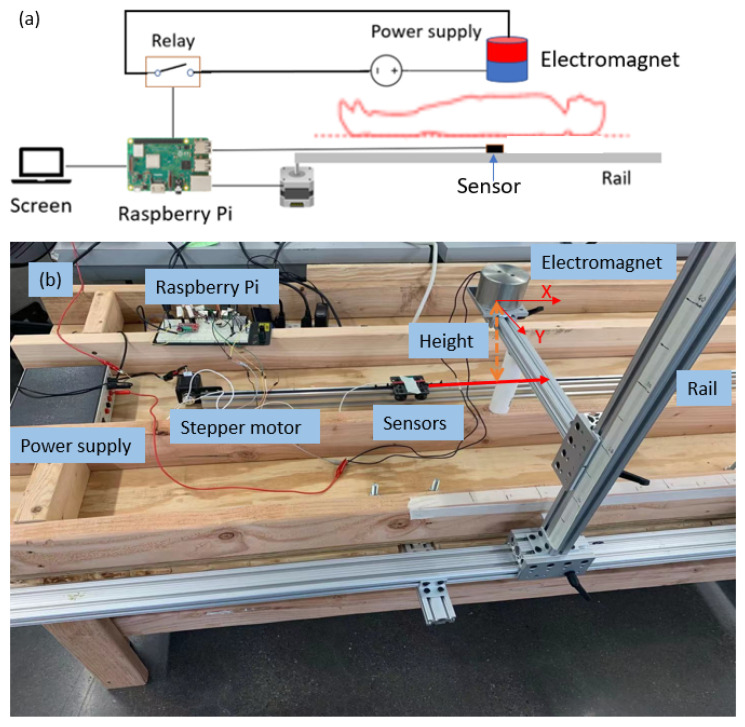
(**a**) The schematic of the system. (**b**) The realization of the system in the lab.

The schematic of the entire system is shown in Figure 7a (the person shown is for reference only—no tests were carried out with human subjects in this work), and Figure 7b shows its realization in the lab. Besides all parts introduced previously, a stepper motor is used to drive the sensor to known locations, which will be used as the ground truth to check the accuracy of the measurement. The motor drives the sensor to move on a straight rail. The rail in Figure 7b simulates the artery, whose direction is aligned in parallel with the *X* axis in the magnet frame. In this simplified condition, the coordinate change along the rail only occurs in the *X* axis component, with the other two components being static. The height between the sensor path and the magnet shown in Figure 7b is related to the thickness of a patient’s torso. As the height increases, the magnetic field strength in the artery decreases, so there is a limit to how high the magnet can be placed. The height is not expected to be greater than 20 cm for the soldier population of interest in this application.

**Figure 8:**
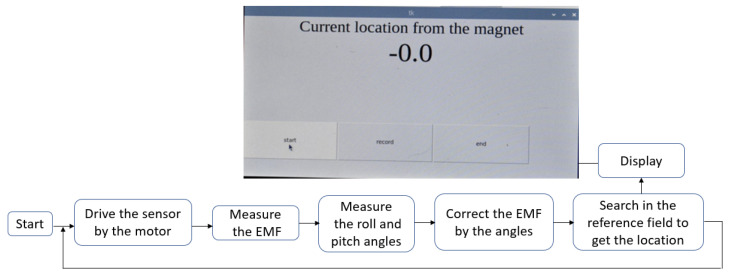
Schematic of the system workflow, including the user interface that shows the sensor’s location relative to the origin.

The first test to be presented involves several tests in which the magnet is fixed at different heights above the sensor. The workflow for each test is shown in Figure 8. The testing range on the rail starts from 15 cm to the left of the magnet (at location −15 cm) and ends at 15 cm to its right (at location +15 cm). The experiment runs by positioning the stepper motor so that the sensor is at location −15 cm, then moving it to +15 cm in 0.2 cm increments, pausing at each increment (at which point a measurement is made). In the magnet frame, this can be expressed by the X component of its coordinate changing from −15.0 to +15.0. This component represents the location of the sensor inside the artery, so it is the most important one in this application. At each pause, the position detection algorithm is executed. Namely, the current to the electromagnet is turned off to update the background magnetic field, the roll and pitch angles are measured to correct the SMF, and the search algorithm is run on the Raspberry Pi to calculate the sensor’s position. The surgeon can read the location from the screen, as shown in Figure 8.

**Figure 9:**
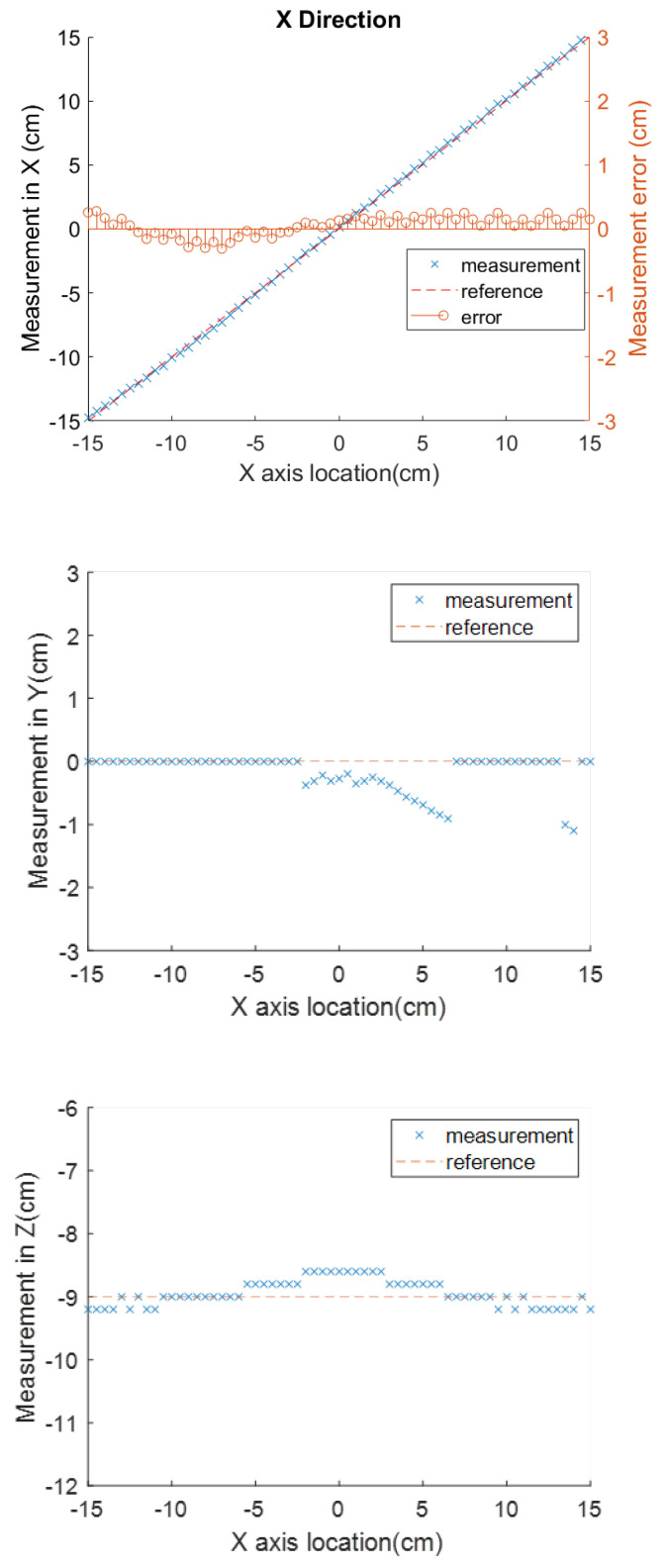
Location measurements along three axes with the magnet height at 18 cm.

The measurement results along the three axes are shown for different heights of the electromagnet in Table 2. For Table 2 and in the rest of this section, the measurement performance will be evaluated by three factors. These are the overall averaged error (AVE), the standard deviation (STD), and the origin error (OE) (where the measurement should be zero as the sensor is right under the center of the magnet, which is the origin of the coordinate system). AVE and STD are metrics of the overall locating accuracy, while OE is the key metric of success in this application. These three factors will be shown in parentheses when mentioned in the text below. For example, the error in the X-axis measurement at a height of 25 cm is (AVE, STD, OE) = (0.36 0.31, 0.00). Based on discussion with the surgeons, the most important error measurement is the *X*-axis position at the point of final placement of the stent, which is designed to be when the sensor is directly under the magnet. The target error limit for this instance is 0.5 cm. For reference, the renal artery, which will be aligned with the uncovered part on the stent upon proper placement, has an average diameter of 0.6 cm [32]. There is also a secondary consideration for stent deployment, which is that the surgeons wish to see a steadily decreasing location value as the stent approaches its target. To provide an indication of the error variation throughout the deployment, we provide the AVE and STD results. While there are no specific targets for these, we wish to keep them below 0.5 cm as well so that there are no confusing fluctuations in the readings that will be displayed to the surgeon. The results generally fall within these targets. For example, a specific test result at the height of 18 cm is shown in Figure 9. In this example, the three evaluation errors in the *X* direction (0.16, 0.08, 0.18) are the smallest among the three directions. In comparison, there is a larger error along the *Y* axis at (0.72, 0.70, 0.57) and the *Z* axis at (0.19, 0.32, 0.40), especially near the beginning of the test (at the left side of the operating range); however, these are not relevant for the surgeon.

### 3.2. Validation of Magnetic Field Interference Cancellation

**Figure 10:**
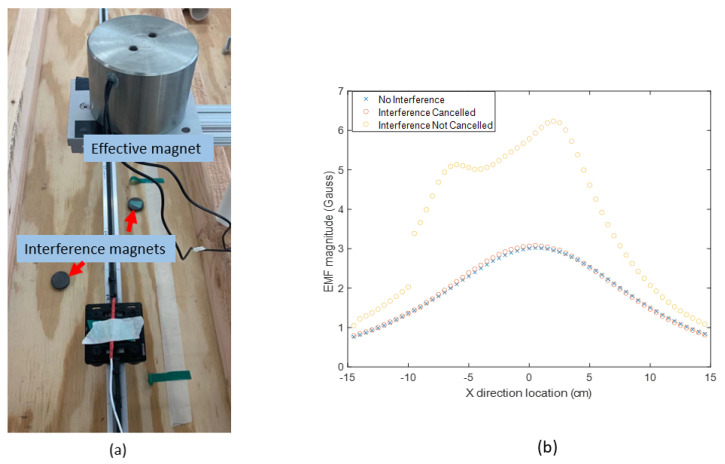
Validation of the disturbance-canceling method. (**a**) Extra magnets introduced as the disturbance sources. (**b**) The effect of the canceling algorithm.

**Figure 11:**
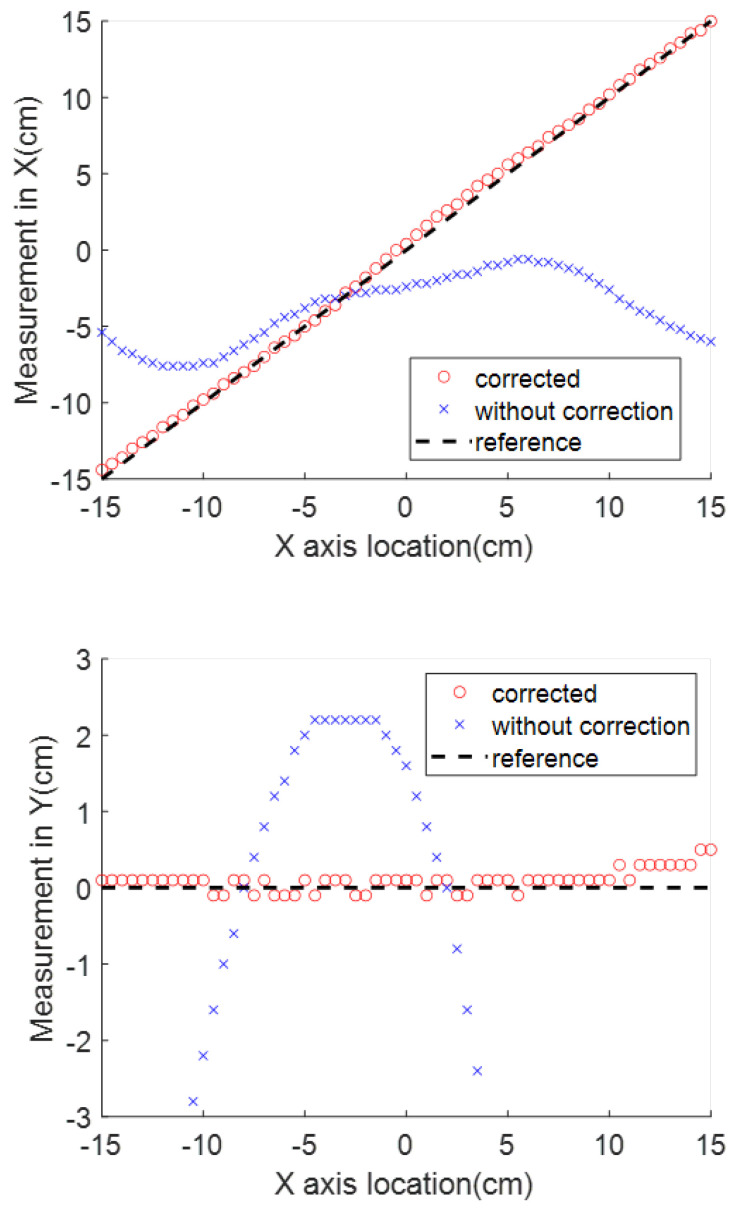

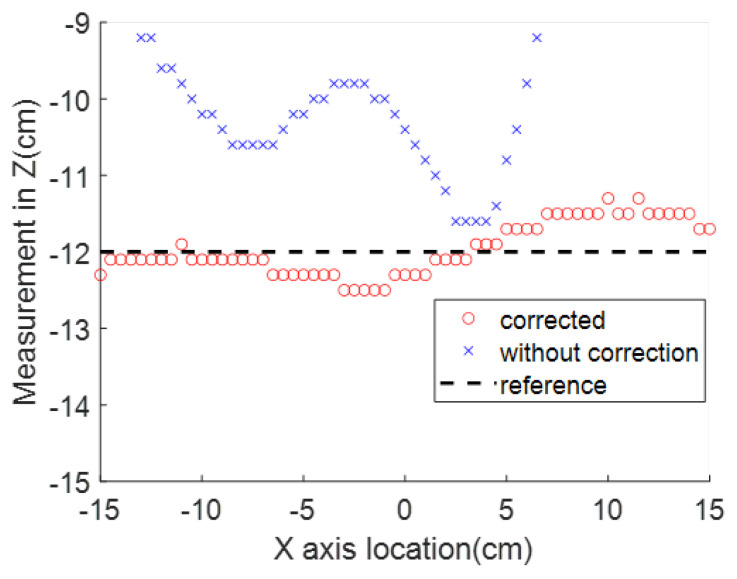
The comparison of location measurements along three axes with and without the disturbance-canceling approach.

Building on the ideal condition test, extra hard iron interference is introduced by placing two permanent magnets at random locations along the moving trajectory, as shown in Figure 10a. To validate the effectiveness of the current-switching method in canceling the hard iron interference, the magnetic field measurement with interference magnets is compared with data from a test in which they are not present. From the comparison shown in Figure 10b, with the interference-canceling method, the maximum deviation is reduced from 3.25 to 0.033 Gauss, and its improvement in location measurement is obvious, as shown in Figure 11. This test shows that the method successfully removes the external distortion and leaves the desired magnetic field to be used for location calculation.

**Figure 12:**
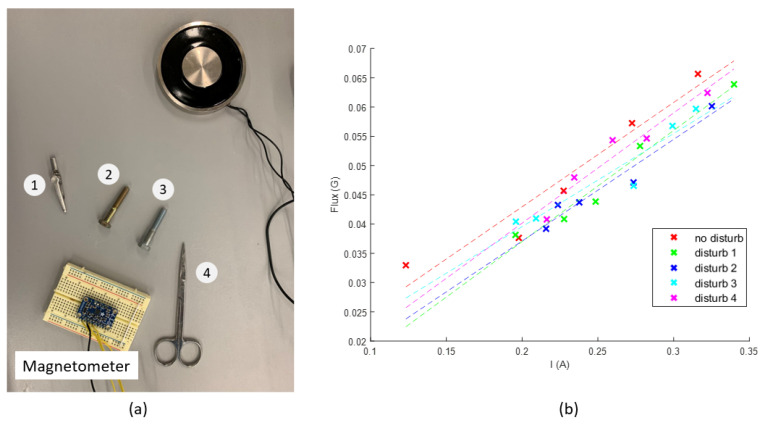
The soft iron interference measurement test. (**a**) Disturbing objects added one after another around the magnetometer. (**b**) The slope values of the curves fitted to all datasets are close.

Next, the test setup to measure the amplitude of soft iron interference from the objects around the sensor is shown in Figure 12a. The soft iron interference is introduced by four ferrous objects placed one after another around the magnetometer in the order shown in the figure; the disturbance term in Equation (8) is changed from ${\vec{V}}_{soft1}$ until ${\vec{V}}_{soft1}+{\vec{V}}_{soft2}+{\vec{V}}_{soft3}+{\vec{V}}_{soft4}$. As introduced before, the magnitude of this term is the slope parameter in the linear relation between *I* and $|{\vec{B}}_{m}|$. The electromagnet and the sensor are kept stationary so that their relative location does not change in the whole process. After each disturbing object is added in, the current is randomly adjusted to five different values and measured by the current sensor. The magnitude of the field is measured correspondingly. A linear curve is determined based on the five measurements using the curve fitting approach. In the result shown in Figure 12b, the slopes of the five experiments are calculated as 0.178, 0.189, 0.173, 0.159, and 0.188. Compared to the first experiment, whose slope is 0.178, the largest difference occurs in the fourth experiment, whose slope is 0.159. The ratio between these two experiments is 0.893, which means at the worst condition, the field measurement is 0.893 times the actual field due to the soft iron disturbances. When the magnetic measurement data from the ideal condition test introduced in Figure 9 are multiplied by the ratio of 0.893, the average location measurement variants in the three directions are (0.29, 0.019, 0.69) cm. In addition, the location variants in the three directions are smallest ((−0.02, 0.019, 0.63) cm) at the target location and largest ((0.6, 0.2, 0.8) cm) at the initial location. The test shows that the soft iron effect from common ferrous objects is not a significant cause of error in this application. If the surgeon only cares about the locating accuracy at the target location in practice, it is reasonable to ignore this problem.

### 3.3. Validation of Angle Measurements with Accelerometer

**Figure 13:**
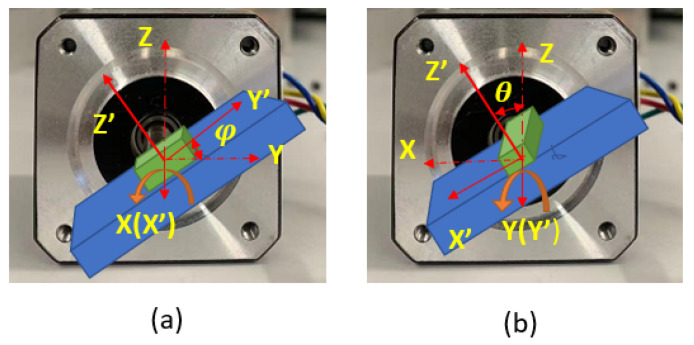
Using stepper motor as the rotation angle reference. (**a**) The sensor’s *X* axis is aligned with the motor shaft to change the roll angle. (**b**) The sensor’s *Y* axis is aligned with the motor shaft to change the pitch angle.

The validity of the tilt compensation algorithm requires that the roll and pitch angles can be measured accurately during stent deployment. This section will validate the roll and pitch angle measurement by an accelerometer with a stepper motor to drive the sensor to known angles. These angles will be compared with the calculated angles through Equations (15) and (16) to validate the function of the accelerometer for angle compensation. The roll and pitch angles are validated separately by aligning each axis of the sensor in turn to the motor shaft, as shown in Figure 13. For roll and pitch angle, 0° is defined when the Z axis is aligned vertically. Clockwise rotation is denoted as positive, and counter-clockwise rotation is denoted as negative. Based on practical experience and due to the confinement of the stent/sleeve/sensor unit as it moves along the narrow and nearly straight artery, the pitch angle will be restricted to a small value, usually less than 15°. It can more freely rotate around the X axis, however, so in this work, the roll angle φ is validated over a larger range compared with pitch angle θ.

The results are shown in Table 3 and Table 4, and they show the effect of rotation on the average locating error with and without the angle compensation. The columns of the tables show the actual angles imposed during the test, the calculated angles based on the accelerometer, the increased *X*-axis average error without the calculated tilt, and finally the *X*-axis error when the measured tilt is compensated. The inclusion of the angle compensation algorithm effectively reduces the locating error, particularly for the cases in which roll angles are introduced. As expected, the error becomes much more drastic at large angles (e.g., over 4 cm for $135^{o}$ of roll or $20^{o}$ of pitch). In all cases, the compensation reduced the errors due to tilt angles to about 0.2 cm for roll and about 0.5 cm for pitch. For comparison, take the magnetic locating test shown in Table 2. When no rotation occurs, the average error in the *X* direction was between 0.16 and 0.36 cm across the 30 cm path.

While it is expected that, in practice (while the stent and sensor are in the blood vessel), the roll angle will have larger variance than the pitch angle, Table 4 shows that a smaller change in pitch causes a larger error in locating. For example, the variation of 10${}^{\circ }$ in pitch causes an error of 2.34 cm, while a 90${}^{\circ }$ variation in roll only creates an error of 0.89 cm. Furthermore, the introduction of pitch angle compensation can exacerbate the error. For example, at 0 degrees of pitch, the *X*-axis error is almost as high as the full allowed error. The reason for this extra error is in the incorrect measurement of the pitch angle itself (e.g., for the true 0${}^{\circ }$ case, the measured angle is −3${}^{\circ }$, which leads to the error). In the static condition, the standard deviation of the noise measurement in each axis of the applied accelerometer is 0.011 m/s${}^{2}$, which causes an angle measurement error of up to 0.06${}^{\circ }$. This suggests the signal noise is not a primary issue. Instead, imperfectly calibrated accelerometer bias could be a greater contribution to error. In conclusion, a more accurate measurement of all three Euler angles is an objective for future research.

### 3.4. Combining All Disturbances

**Figure 14:**
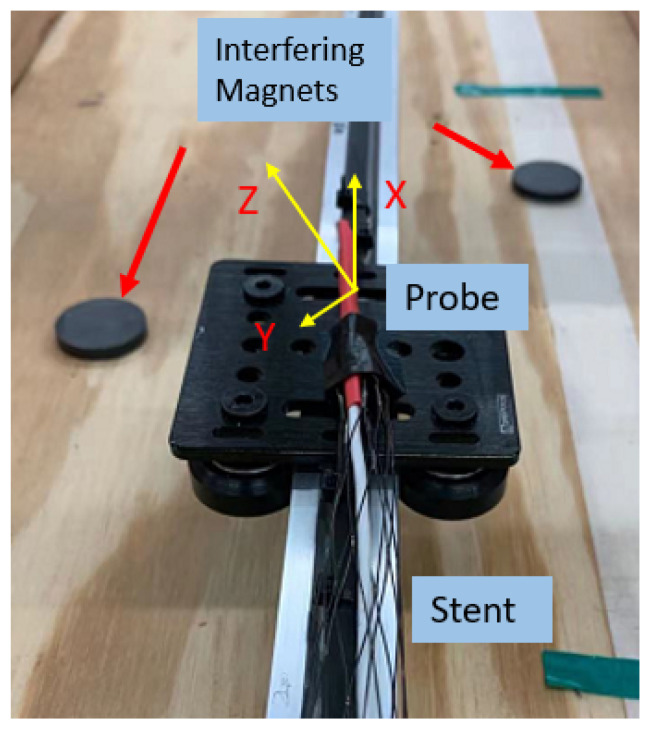
Experiment setup with all interfering factors.

**Figure 15:**
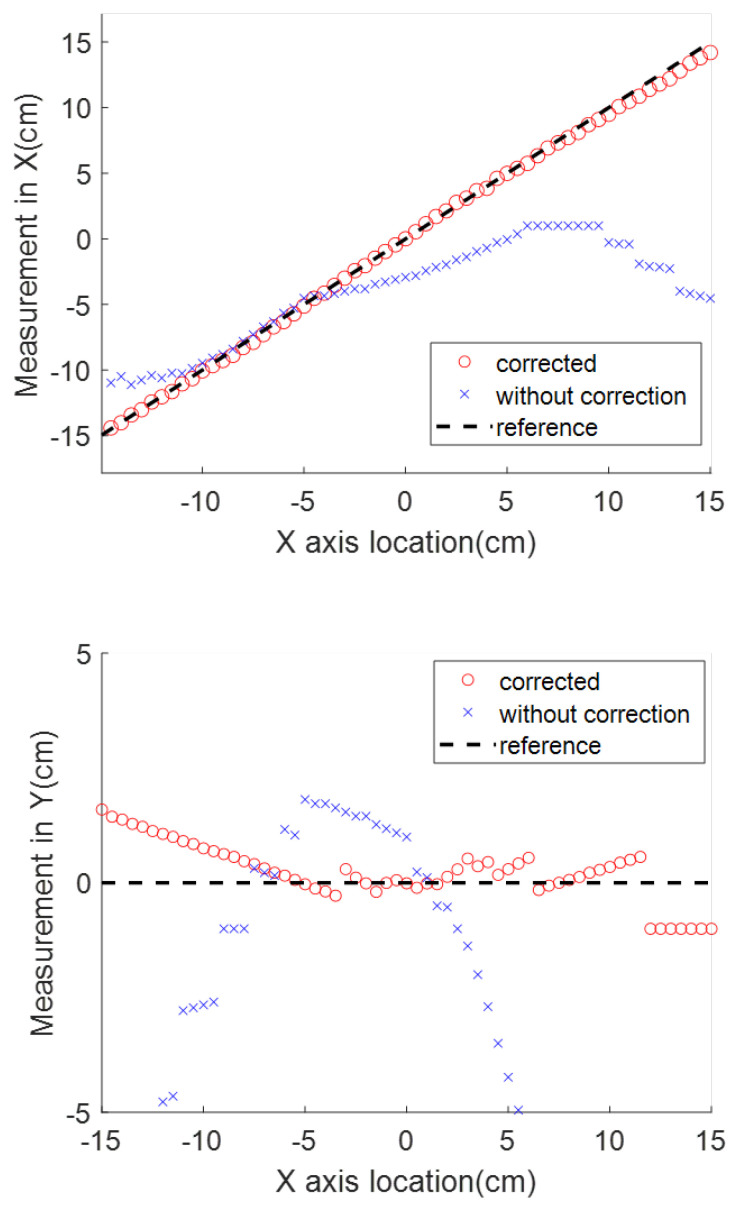

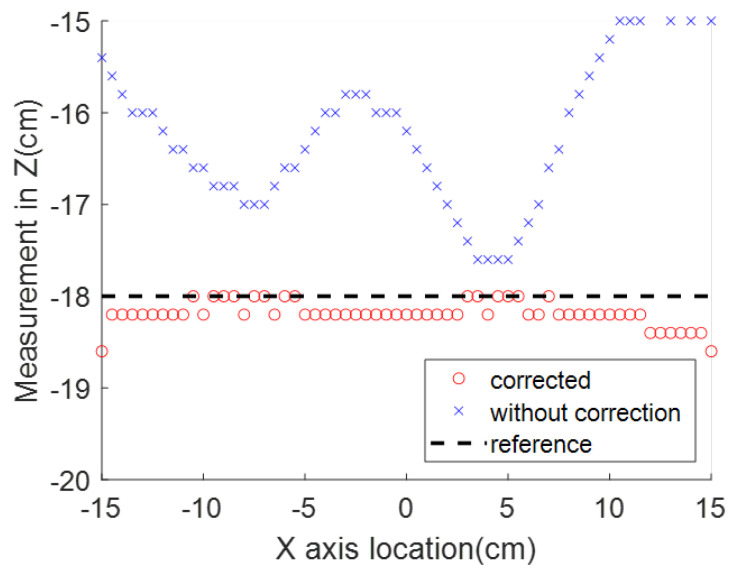
Comparison between the measurement with and without all the disturbance correction procedures.

Finally, a test was designed in which all of the above-mentioned disturbances were included. The test setup is shown in Figure 14. The magnet source is placed 18 cm vertically above the sensor. The sensor’s *Z* axis is not aligned vertically, due to a roll angle error. The detecting probe is attached to the stent. Two extra magnets are placed along the rail as interference. The results are shown in Figure 15 for both the case with no compensation and the case in which the compensation methods described above are used. Compared with the measurement using raw data, which are the dots in Figure 15, the correction methods can greatly reduce the deviation. When the correction methods are applied, the error along the *X*, *Y*, and *Z* directions, represented by the averaged error, standard deviation, and zero point error, are (0.24, 0.22, 0.01), (0.50, 0.43, 0.01), and (0.18, 0.13, 0.2), respectively. Compared with the results achieved under ideal conditions, shown in Table 2, there is increased error and standard deviation in all directions. In the *X* direction, the averaged error increased from 0.16 cm to 0.24 cm. Of particular interest for the application, the results at the target location are still well within the desirable range, as the error at the center point is only 0.01 cm.

## 4. Conclusions and Limitations

It was the goal of this work to develop a simple system for locating a stent within a human body that could be rapidly deployed in the field, particularly in a battlefield situation. This paper shows that the goal may be achieved with a magnet-based localizing system. The system’s robustness was tested against various disturbances and nonidealizations. The overall average error of 0.29 cm and the zero point error of 0.10 cm are well within acceptability for the application of interest. Most importantly, the measurement when the sensor locates vertically under the magnet is especially accurate (near the ideal situation), which guarantees accuracy when it is used as the location landmark.

However, there are still several limitations that can lead to improvements in the system. First, within the magnetic map created by a single cylindrical magnet, the accuracy is especially good along the radial directions but less accurate along the other two axes. Second, the measurement reliability also increases when the sensor is closer to the magnetic field source, where the random noise effects become insignificant as the desired field magnitude grows larger. Third, the pitch angle measurement must be very accurate if it is to be used in angle compensation. Finally, the system has to be validated in the conditions that simulate the real in vivo environment, which might cause unexpected disturbances and bring requirements for the safety of the patient. Overcoming these obstacles will further improve the value of the technique for locating an object in the human body.

## Figures and Tables

**Table 1:** Electrical parts used in the system.

Part Name	Manufacturer	Model Number
Controller	Raspberry Pi	Raspberry Pi 4 Model B
Magnetometer	MEMSIC	MMC5603NJ
Accelerometer	MEMSIC	MXC400xXC
Current sensor	Adafruit	INA219
Digital relay	P&B Brand	RTE24005F
Electromagnet	BUNTING	BDE-3020-12
DC power supply	MORNSUN	SE-350-24

**Table 2:** Measurement error evaluation at different heights. The three values in each block represent the average error, standard deviation of the error, and the error when the sensor is under the center of the magnet.

Height (cm)	Errors in X (cm)	Errors in Y (cm)	Errors in Z (cm)
18	0.16, 0.08, 0.18	0.72, 0.70, 0.57	0.19, 0.32, 0.40
21	0.22, 0.11, 0.15	0.85, 0.67, 0.81	0.15, 0.12, 0.00
25	0.36, 0.31, 0.00	1.10, 0.71, 1.20	0.57, 0.79, 0.00

**Table 3:** Roll (φ) measurement and its effect on locating.

Motor Setting Reference (Degree)	φ Measurement (Degree)	Error without Correction (cm)	Error with Correction (cm)
−135	−134.2	4.16	0.22
−90	−85.1	0.89	0.23
−45	−47.7	0.45	0.21
0	1.6	0.16	0.23
45	46.8	0.34	0.23
90	85.3	0.66	0.23
135	139.9	2.54	0.22

**Table 4:** Pitch (θ) measurement and its effect on locating.

Motor Setting Reference (Degree)	θ Measurement (Degree)	Error without Correction (cm)	Error with Correction (cm)
−20	−22	4.61	0.47
−10	−12.5	2.34	0.56
0	−3	0.22	0.47
10	7	2.34	0.34
20	16.5	4.56	0.67

## Data Availability

Not applicable.
